# Positive vs. Negative: The Impact of Question Polarity in Voting Advice Applications

**DOI:** 10.1371/journal.pone.0164184

**Published:** 2016-10-10

**Authors:** Bregje Holleman, Naomi Kamoen, André Krouwel, Jasper van de Pol, Claes de Vreese

**Affiliations:** 1 Uil-OTS, Utrecht University, Utrecht, The Netherlands; 2 TiCC, Tilburg University, Tilburg, The Netherlands; 3 Social-Cultural Sciences, Free University, Amsterdam, The Netherlands; 4 Kieskompas bv., Amsterdam, The Netherlands; 5 ASCoR, University of Amsterdam, Amsterdam, The Netherlands; Eberhard-Karls-Universitat Tubingen Medizinische Fakultat, GERMANY

## Abstract

Online Voting Advice Applications (VAAs) are survey-like instruments that help citizens to shape their political preferences and compare them with those of political parties. Especially in multi-party democracies, their increasing popularity indicates that VAAs play an important role in opinion formation for citizens, as well as in the public debate prior to elections. Hence, the objectivity and transparency of VAAs are crucial. In the design of VAAs, many choices have to be made. Extant research in survey methodology shows that the seemingly arbitrary choice to word questions positively (e.g., *‘The city council should allow cars into the city centre’*) or negatively (*‘The city council should ban cars from the city centre’)* systematically affects the answers. This asymmetry in answers is in line with work on negativity bias in other areas of linguistics and psychology. Building on these findings, this study investigated whether question polarity also affects the answers to VAA statements. In a field experiment (*N* = 31,112) during the Dutch municipal elections we analysed the effects of polarity for 16 out of 30 VAA statements with a large variety of linguistic contrasts. Analyses show a significant effect of question wording for questions containing a wide range of implicit negations (such as ‘*forbid*’ vs. ‘*allow*’), as well as for questions with explicit negations (e.g., ‘*not*’). These effects of question polarity are found especially for VAA users with lower levels of political sophistication. As these citizens are an important target group for Voting Advice Applications, this stresses the need for VAA builders to be sensitive to wording choices when designing VAAs. This study is the first to show such consistent wording effects not only for political attitude questions with implicit negations in VAAs, but also for political questions containing explicit negations.

## Introduction

VAAs are online tools that help citizens to shape their political opinions and to figure out which parties or candidates are most in line with their own individual position, in order to make a vote choice. After reacting to a range of statements, the user’s answers are compared to the positions of all the political parties in a certain election. This way, VAAs offer a concise way to obtain information about the issues and party positions in a particular election in a time-efficient and user-friendly way (e.g. [1]).

The popularity of VAAs has increased over recent years and has spread from one country to the other. Currently, about 10% to 40% of the electorate in many European countries uses a VAA in the weeks prior to Election Day [2]. Because so many voters use VAAs, there is a serious possibility for them to have an impact on individual users as well as on public opinion at large. VAAs help individual users to learn about the important issues in an election and help to understand their own position relative to the parties’ positions, but they may also affect individual’s perceptions due to issue selection, statement wording or the presentation of the advice. Furthermore, they might play an agenda-setting role in the political debate ahead of elections. VAAs can hence be viewed as interactive platforms with a reach comparable to mass media. This means balancing the demands of the market with the democratic requirements of modern states—rather than just being informers of the discussion, VAAs are also constructing the debate, and fuelling it [3].

Therefore, a wide range of scholars including political scientists, philosophers, and public opinion researchers are studying VAAs as a societal phenomenon and as an individual decision making tool: what does the design of VAAs reflect about our society’s views on democracy and public opinion formation (e.g., [4])? At the level of individual opinion formation, research focuses on how and to what extent the use of these tools affects citizens’ electoral behaviour (e.g., [5], [6], [7]); and on the extent to which design choices VAA builders make in constructing their tool affect the answers to the VAA statements, as well as the voting advice. A variety of such design choices has already shown to influence the voting advice people receive; e.g., the selection of issues and number of statements (e.g., [8], [9]), the way the match between users and each party is presented, and the algorithm used to calculate this match (e.g., [10], [11]). However, with the exception of a descriptive corpus study focusing on question wording choices and content of VAA statements [12], there is a paucity of research on the formulation of statements in VAAs and its effect on VAA answers (and subsequently, also on the voting advice).

In the area of survey methodological research, extensive work has been done on the way question wording (unintendedly) affects the answers to attitude and opinion questions. As VAAs consist of survey-like opinion statements, this field of research is critical for VAA design choices. One wording characteristic that has considerable impact on the answers obtained in attitude surveys, is the positive versus implicit negative wording of political attitude questions or statements, such as ‘Cars should be *allowed in* the city centre‘ and ‘Cars should be *banned from* the city centre‘. Building on to this work in surveys (e.g., [13], [14]), this paper presents a randomized field experiment on a real-life Voting Advice Application as developed for the municipal elections in a large Dutch city (Utrecht). We assessed the impact of positive versus negative question wordings (valence framing) in VAA questions on the answers obtained. In addition to investigating this for implicit negations (e.g., ‘*forbid*’ vs. ‘*allow*’), we also zoom into the effects of the use of explicit negations (e.g., ‘*not*’). Furthermore, we consider political sophistication as a moderator of these effects. The current study is the first to investigate the effect of question polarity of a wide range of contrasts on the answers in Voting Advice Applications. This gives us a sense of how subtle wording variation affects political attitudes, both through reading and answering the questions and of how this relates to levels of political sophistication in this context of motivated use.

### Wording effects for contrastive questions in attitude surveys

The first study in which an effect of positive versus negative question wording choice was ‘discovered’, dates back to the 1940s when Rugg [15] conducted a survey experiment concerning the opinions towards free speech. One random half of the sample was asked whether they felt ‘*public speeches against democracy*’ should be ‘*forbidden*’, and the other half was asked whether they thought, ‘*public speeches against democracy*’ should be ‘*allowed*’. The results indicated that fewer respondents were willing to endorse the ‘*forbidding*’ of those speeches (54%) compared to the number of respondents who indicated ‘*no*’ to the ‘*allow*’-version of the question (75%). Hence, respondents seemed to hold more favourable attitudes towards the attitude object ‘*public speeches against democracy*’ when the question was worded with ‘*forbid’* compared to when it was worded with the verb ‘*allow*’.

This fundamental insight sparked a series of studies attempting to replicate and extend the forbid/allow effect for questions on a large variety of political issues, ranging from *speeches against democracy* to *X-rated movies*, from the *use salt on slippery roads in the winter* to *smoking in public places* (e.g., [16]; [17]; [18]; [19]). Some studies replicated the forbid/allow asymmetry found by Rugg in 1941, (e.g., [17]; [18]), whereas others found no effect of question wording in some questions (e.g., [16]), or even an effect in the opposite direction (e.g., [19]).

Despite these heterogeneous results, a meta-analysis revealed that the forbid/allow asymmetry can be generalized across the large variety of political yes/no-questions reported in the literature [20]. Overall, respondents are substantially more likely to report more favourable attitudes to the negative '*forbid’* wording, i.e. to be more prone to answer ‘*no*’ to ‘*forbid’*-questions than ‘*yes*’ to ‘*allow’*-questions. This overall effect on the answers is often explained by the tone of wording of ‘*forbid*’ versus ‘*allow*’: “The verb ‘*forbid*’ sounds harsher and may therefore be more difficult to endorse, whereas ‘*allow*’ in some contexts might seem to encourage deviant behaviour and therefore may invite opposition” ([16]: 280).

Subsequent research shows that the forbid/allow asymmetry is not restricted to binary yes/no-questions, but also occurs for 7-points scale forbid/allow questions [21]. In addition, the asymmetry seems to be generalizable to some other implicit negations besides ‘*forbid’* vs. ‘*allow’*: to some other contradictory word pairs such as ‘*restrict’* vs. ‘*leave free’* [18]) as well as to a large range of contrary word pairs, such as ‘*good’* vs. ‘*bad’*([14]; [22]; [23]). In sum, the picture emerging from these studies is that implicit negative questions lead to more ‘*no’*-answers or ‘*disagree’*-answers as compared to the number of ‘*yes’*-answers or ‘*agree’*-answers to their positive counterparts.

Hence, generally speaking, the answers to negative questions seem to reflect a more positive evaluation of the attitude object. This leads to the conclusion that the forbid/allow asymmetry is probably not just caused by the tone of wording of ‘*forbid*’ vs. ‘*allow*’, but rather by a general *negativity bias* [24], [25]. Negativity bias refers to the notion that things of a more negative quality (such as unpleasant events, or feelings) have a larger impact on people’s evaluations and processes than positive or neutral things. From this, it follows that positive and negative contrasts do not carry the same weight: positives are used more frequently and reflect more moderate meanings compared to their (more extreme) negative counterparts. Due to this extremity, it may be more difficult to endorse a negative question with an agreeing answer than to answer ‘*no*’ to the equivalent positive question.

Although the effect of implicit negatives, and most specifically the forbid/allow wording variation, has been quite thoroughly investigated within the area of survey methodology, it has never been studied within the context of VAAs. It is relevant to do so, because a wide range of implicit contradictory verbs, including ‘*forbid*’ and ‘*allow*’, are used in the context of the political policy issues addressed in VAAs. And even though VAAs look like surveys in many ways, they are not necessarily the same. People use VAAs for different reasons compared to filling out surveys: they might be quite motivated to respond in order to obtain a personalized voting advice, and therefore wording effects arising in surveys do not necessarily occur in VAAs as well.

While the effects of implicit linguistic contrasts have been studied extensively in surveys, the effect of explicit negations has received only very little attention. This is probably due to the fact that the use of explication negations in attitude surveys is generally advised against, as such question wordings would lead to confusion as to the meaning of the answers, because answering ‘*no*’ or ‘*disagree*’ to a negative question would result in a double negation (e.g., [26]). Explicit negatives are quite widely used in a VAA context, however ([27]). Hence, it is feasible to include an investigation into the effects of these wording variations in this context.

Broadening our focus not only to the effect of a wider range of *implicit* negatives but also to *explicit* ones, will lead to more general conclusions on negativity bias in political attitude questions. Furthermore, it will generalize the occurrence of these wording effects beyond political surveys into the context of Voting Advice Applications.

### Political sophistication as a moderator for wording effects

An important issue is whether question polarity affects the answers across-the-board, or whether variation in the size of the effect can be systematically related to respondent characteristics. Generally, in literature about information processing, dual-route theories such as Elaboration Likelihood Theory (ELM, [28]) have proposed that people who are interested or personally involved in an issue, tend to process information about that issue more deeply. This means they are likely to scrutinize the issue addressed in the survey question, whereas people who are less involved or interested tend to perform shallow or heuristic processing. By doing so, this latter group will be more susceptible to superficial characteristics of the way the information is conveyed (e.g., wording, or source credibility) and more likely to use relatively general rules of thumb to judge the information (e.g., if many vote for party x, it is likely to be a good party).

In a similar way, in the area of survey methodology, Krosnick [29] proposes that respondents deal with the high cognitive demands of survey answering by ‘satisficing’: the less motivated respondents are to fill out the survey, the stronger their satisficing behavior, ranging from using heuristics to reach an answer, to just providing any answer that seems plausible. This line of reasoning has led to a great deal of research investigating the relations between motivation, interest, personal relevance, and attitude strength [30], leading to the prediction that “…it is weak attitudes (barely attitudes at all) that are susceptible to response effects; strong attitudes, in contrast, provide an anchor that is substantial enough to withstand the effect of subtle question variations” ([31]:108).

However, using meta-cognitive measures of attitude strength, such as reported certainty about one’s own attitude, empirical evidence in extant survey research is very heterogeneous: in studies on the forbid/allow asymmetry, for example, often the wording effect can be explained by (indicators of) attitude strength, but equally often attitude strength is found unrelated to the asymmetry. For attitude intensity, Krosnick and Schuman [32] even find an interaction effect in the direction opposite to expectations: larger wording effects for respondents with more intense attitudes—which is in line with results by [31] who find larger wording effects for respondents who report the issue to be important or hold intense attitudes. Holleman [20] finds no relation between the forbid/allow asymmetry and several meta-attitudinal strength measures across a set of questions, but [17] show that if respondents who have answered to be “indifferent “about a certain issue are excluded from the analyses, the asymmetry will disappear. And Narayan and Krosnick [33] find the asymmetry can be mainly attributed to lower educated respondents.

Comparable to theorizing in social psychology and survey methodology, research in political decision making has shown that a voters’ degree of political sophistication affects the way he or she processes and organizes political information (e.g., [34]), and the susceptibility to contextual factors (e.g., [35]). Political sophistication is related to political interest (which is connected to general interest often measured in the attitude strength literature, but is also known to be strongly related to levels of political knowledge, e.g., [36], [37]) as well as to education (found to moderate the forbid/allow asymmetry in [33]). Furthermore, political sophistication is relevant in this context of political VAA statements. Therefore, we will use political sophistication here to investigate the extent to which certain groups of VAA users are more sensitive to variation in the wording of our political questions.

### Research questions and hypotheses

The current study adds an important large-scale field study to the existing body of relatively isolated small-scale experiments on opinion survey wording effects in the context of a VAA. A substantial number of questions was manipulated by using a range of implicit and explicit negative question wordings on a variety of question topics. This allowed us to assess the overall effect of each type of question wording, as well as the variation of this effect across different questions. Based on previous research on this topic in the area of survey research, we hypothesize

- *H1a*: *VAA users will more often disagree with negatively worded questions containing implicit negatives than agree with equivalent positive ones*.
  In addition, in the current research we explore
- *H1b*: *VAA users are likely to disagree more often with questions containing explicit negations than to agree with their positive counterparts*.
  Furthermore, we will analyse the moderating effect of political sophistication, hypothesizing
- *H2*: *the effect of question polarity is smaller for VAA users who are politically sophisticated than for VAA users who are relatively less sophisticated*.

## Method

### Design and materials

In the weeks preceding the 2014 municipal elections, we carried out a field experiment in collaboration with Dutch VAA builder *Kieskompas* (Election Compass) and the Utrecht city council. The project description was approved prior to fielding the study by Utrecht University, by the Dutch Science Foundation (NWO) and by the Utrecht City Council.

Following regular procedures including consultation rounds with all political parties (see [34]), *Kieskompas* and the Utrecht city council constructed a VAA for citizens in Utrecht (the fourth largest city in the Netherlands). This resulted in a voting advice tool very comparable to VAAs in other municipalities, consisting of 30 political opinion questions. After this original benchmark version had been construed, our research team developed four additional experimental versions of the VAA. All political parties, the Council Presidium, and VAA builder *Kieskompas* approved of the construction and fielding of these extra VAA versions. Participation in the VAA was voluntarily and anyone could stop using the tool at any point in time.

**Fig 1:**
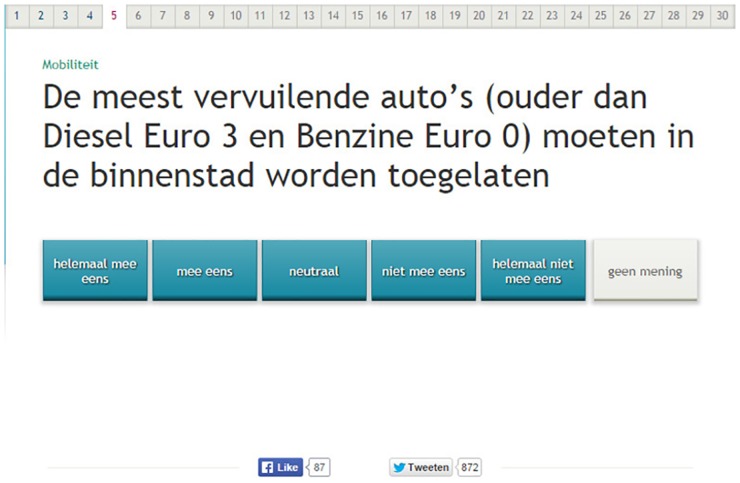
Example of a positive question version as presented on screen. Legend: *The question reads* “The most polluting cars (older than Diesel Euro 3 and Gas Euro 0) should be allowed in the city centre.”

**Fig 2:**
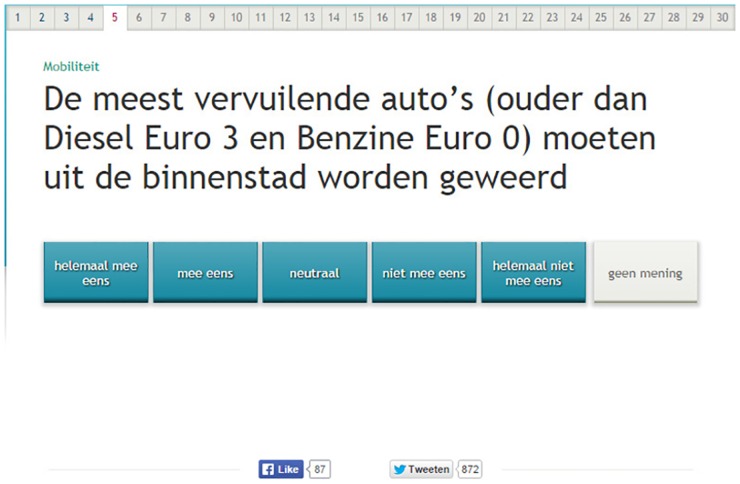
Example of a negative question version as presented on screen. Legend: *The question reads* “The most polluting cars (older than Diesel Euro 3 and Gas Euro 0) should be banned from the city centre.”

In two experimental versions, the polarity of 16 questions was manipulated. Fig 1 shows what the VAA statements looked like. The questions consisted of two types of contrasts. Six negative question versions contained an implicit negative (e.g., ‘*allow’* versus ‘*ban’*, see Fig 2), 10 questions contained an explicit negative (e.g., ‘*not’*, as in ‘The municipality *can cut down* / *can**not* *cut down* on social work’). A total of 13 out of the 16 statements were manipulated following a 2 (question polarity: positive or negative) x 2 (heading above the question: left-wing or right-wing) design; in addition, there were 3 questions for which only the polarity of the question (positive versus negative) was varied. In the present article, we only report about the effect of question polarity because the effect of question polarity statistically interacted with the effect of the heading manipulation for only 2 out of the 13 questions, which is to be expected based on chance.

The manipulated questions were about a wide range of political issues ranging from taxes to circuses with wild animals, from cuts in the art budget to mobility issues; and about the continuation of existing policies as well as the development of future ones. An overview of all manipulated questions is provided in S1 Appendix. As can be read from this S1 Appendix, each experimental VAA version contained both positive and negative question wordings, and both implicit and explicit negatives.

### Measures

Users of the VAA were asked to what extent they agree with each statement on a five-points scale ranging from ‘*completely agree*’ to ‘*completely disagree*’. The dependent variable is formed by these mean answers, recoded in such a way that a higher score indicates a more positive evaluation of the attitude object. Non-substantive answers are excluded from the analyses.

Political sophistication can be assessed in various ways. Some [35] pose a number of questions about political interest and political participation; others measure political knowledge by counting correct responses to a series of knowledge questions [38]. In Voting Advice Applications, it is important to avoid the impression with users that the tool can only be used if one has a sufficient level of political knowledge or participation. Therefore, we used a measure of political sophistication suggested by [36] and [39], combining a question about political interest with a measure of educational level into an additive index of political sophistication.

Before answering the VAA statements, about 70% of all VAA users indicated on a five-point scale to what extent they are interested in politics, ranging from ‘*a very high interest*’ to ‘*a very low interest*’. Also we asked about their highest finished degree of education, followed by a 7-category scale and a ‘*don’t know or don’t want to tell*’. In order to combine both scales into one additive measure in which each measure receives an equal weight, we created two new variables with an equal number of scale points. We chose to recode education into 3 scale points, similar to the classification used by CBS (the Dutch Central Bureau of Statistics), and therefore recoded political interest into 3 scale points also, and then combined both variables into an additive sophistication measure with a 5-points scale (ranging from 2–6).

### Participants

The city of Utrecht in 2014 consisted of 328,165 inhabitants at the time of the study. About 80% (258,087 persons) was eligible to vote. This latter group is the VAA target group. *Kieskompas Utrecht* was online during one month (February 18, 2014 through Election day: March 19, 2014), during which 41,505 people accessed the website. This number suggests that roughly 16% of the target group was reached by the VAA. It is impossible to tell whether these 41,505 are all unique users. Different people may have accessed the tool from one and the same IP-address, but some citizens may also have used the tool twice, or more. This does not seem to be a problem for the internal validity of this study however, as double usage of the tool is neither likely to be high, nor related to a systematic overestimation or underestimation of the question wording effects.

The 41,505 visitors of the VAA website were randomly assigned to either the benchmark version, or to one of the four experimental VAA versions. In the current study, we focused on the VAA users who were assigned to one of the experimental versions of *Kieskompas*. VAA users who were directed to the original benchmark version (*N* = 7,646), were excluded from the analyses as this version differed from the four experimental versions in a number of ways. Excluding the 20% who was randomly assigned to the benchmark version, leaves an 80% random sample from the population of *Kieskompas* users in this election for our experiment (*N* = 33,859).

We only took into account in our analyses those VAA users who were 18 years or older (and hence eligible to vote), who took longer than 2 minutes to fill out all 30 statements, and who did not show straight-lining behaviour (i.e. report the same answer to each and every statement). The resulting participant group consisted of 31,112 *Kieskompas* users.

Prior to answering the 30 VAA questions, *Kieskompas* users were asked some demographic details, which about 70% of the VAA users supplied. About half of the *Kieskompas* users who filled out their details are female (50.7%), with a mean age of 37.3 years old (*sd* = 13.8). The VAA-users are fairly highly educated (the median category is ‘*higher educational vocation or university bachelor*‘). The high education group (completed BA or MA degree) contains 17,520 VAA users, and the group in between consists of 3,856 people. With regard to political interest, a mean score of 3.3 on a 5-pointsscale (*sd* = 0.83) was obtained, indicating our VAA users are moderately interested in politics. When re-grouped into three interest groups, the low interest group (scale points 1 and 2) consists of 2,842 VAA users, the medium group (scale point 3) consists of 10,292 people, and the high interest group (scale points 4 and 5) consists of 8,860 VAA users.

To check the internal validity of our study, a randomization analysis was carried out to control whether respondents were equally distributed over the four *Kieskompas* versions with respect to demographic variables, which indicated that there is no reason to assume that sampling error caused differences in the mean answers to the manipulated questions between versions. Users in the different experimental versions were shown to be comparable with respect to sex (*χ*^2^ (3) = 2.90; *p* = .41), education (*χ*^2^ (3) = 11.90; *p* = .85), age (*F* (3, 22207) = 1.58; *p* = .19), and interest in politics (*F* (3, 21990) = 0.34; *p* = .80).

In addition, we checked how our sample compares to the full Utrecht electorate. Our participants are fairly highly educated. This is in line with the general Utrecht electorate: of the population aged 15–65 about 50% is in the category higher educational vocation or university bachelor (according to the Central Bureau of Statistics, CBS 2014). In terms of gender and age, as registered in the “Municipal Basic Administration” (Gemeentelijke Basis Administratie, GBA), the Utrecht electorate consists of slightly more women (52.1%) than men (47.9%), and the mean age of eligible voters is 42.6 (*sd* = 17.5). Hence, in comparison to the full electorate, our sample is younger (*t* (22210) = 57.73; *p* < .001) and consists of slightly more males (*χ*^2^ (1) = 17.7; *p* < .001). VAA users, however, are typically more often male and a little younger than the average voter (for an overview of the literature, see [40]), so our sample of *Kieskompas* users does seem to be an accurate reflection of the general population of VAA users.

### Analyses

To test our hypotheses several multi-level models were used (cf. [41], [42]). The first model estimates the main effect of question polarity on the mean substantive answers across the group of 6 questions containing implicit negations, and separately in a second model for the set of 10 questions containing explicit negations. Non-substantive answers (‘no opinion’ answers) are excluded from this analysis.

In the fixed part of both these models, the mean answer is estimated for positive questions. Subsequently, it is calculated how the mean answer for negative questions deviates from this mean. Hence, our models are an example of an additive multi-level model [43]. The random part of the models accounts for the hierarchical structure of the data. As each respondent in our sample (*N* = 31,112) answered various manipulated questions containing an implicit linguistic contrast (*N* = 6), or containing an explicit linguistic contrast (*N* = 10), each observation is nested within respondents and items at the same time. To account for this, the models distinguish between-item variance (e.g., because an opinion statement about the one issue elicits more positive evaluations than a statement about another issue), and between-respondent variance (e.g., because one respondent provides more positive evaluations than another respondent does)–as well as a residue of error variance. Hence, cross-classified models are in operation ([41], [42]). Appendix 1 supplies a mathematical representation of these models.

As a second step, we examined to what extent wording effects for contrastive questions can be explained from variation in political sophistication within the population of VAA users. Using the continuous 5-point scale variable Political Sophistication, we added parameters to our two initial multi-level models to assess both the main effect of political sophistication and the interaction between political sophistication and question polarity. The annotation of this more complex model can also be found in Appendix 1. Please note that for this latter analysis only those respondents were included who answered both the question on political interest and on education (*N* = 21,881).

## Results

### The effect of question wording

A comparison of the main effect of question polarity across questions and respondents revealed an overall effect of question polarity for the set of questions with an *implicit* negation (*z* = 11.6, *p* <.001). As can be seen in Table 1, this effect is in the expected direction based on the literature: implicit negative questions lead to more positive evaluations of the attitude object.

**Table 1:** Mean substantive answer for positive and implicit negative questions.

	Positive wording	Negative wording
MEANS	3.03	3.08
VARIANCES		
s^2^ questions	0.15	0.15*
s^2^ persons	0.00	0.00*
s^2^ error	1.28	1.28*

* In our model, all variances are estimated simultaneously for positive and negative questions (see Appendix 1).

In terms of effect sizes, the mean differences are small compared to the between question variance: Cohen’s *d* is .13. Another way to classify the effect, is in terms of absolute scale points: 1 out of 20 users shows one scalepoint difference between positive and implicit negative questions.

The effect varies strongly between questions: using the standard deviation between questions, we can see that the wording effect for 80% of the questions lies between -.59 and +.69 scale points, which means that it is also likely for a question with implicit negations not to affect the answers at all, or to show an effect in a direction opposite to expectations—even though the mean overall effect is in line with theoretical expectations.

We ran a comparable model for the set of 10 questions with an *explicit* negation. This analysis also showed an overall mean question wording effect for explicit negative questions (*z* = 5.25, *p* < .001). As shown in Table 2, the wording effect of questions with an explicit negation is in a similar direction compared to the effect for implicit negatives: a significant tendency to evaluate an attitude object more positively when explicit negative questions are asked. Again, we see a large variation in the effect: for 80% of the questions, the effect of explicit negative questions ranges from -.73 to .78 scale points.

**Table 2:** Mean substantive answer for positive and explicit negative questions.

	Positive wording	Negative wording
MEANS	2.98	3.00
VARIANCES		
s^2^ questions	0.21	0.21*
s^2^ persons	0.039	0.039*
s^2^ error	1.211	1.121*

* In our model, all variances are estimated simultaneously for positive and negative questions (see Appendix 1).

Overall, the effect for explicit negatives is significant, but very small: Cohen’s *d* across questions is .05 when compared to the between question variance

### Political sophistication as a moderator for the effect of question wording?

**Fig 3:**
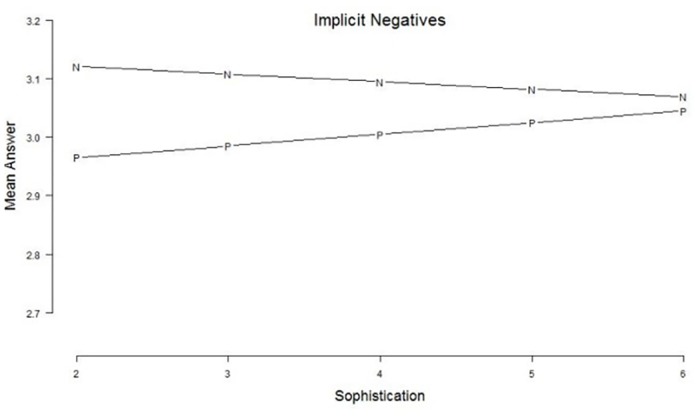
Political sophistication as a moderator of the implicit negatives. *Legend*: The answers are expressed on a 5-points scale ranging from a negative (1) evaluation of the attitude object to a positive evaluation (5). Sophistication is an additive scale combining education and political interest, expressed on a 5 points scale from low sophistication (2) to high sophistication (6).

It is often hypothesized that respondents who hold stronger attitudes towards an issue are less likely to be affected by superficial wording variation. We investigated whether this is the case in our data by modeling the moderating effect of political sophistication on the wording effect for positive versus negative questions (*N* = 21,811). For our set of 6 implicit negative questions we find a significant interaction effect between question wording and sophistication (*z* = 4.7, *p* <.001): the higher the political sophistication of our VAA users, the smaller the difference in mean answers between positive and implicit negative questions (see Fig 3). Again, we computed the effect sizes for the differences between positive questions and implicit negative questions, this time for each sophistication group separately. By relating the mean difference to the question variance, we get a Cohen’s *d* which is .39 for the lowest sophistication level. The effect sizes dampen when reaching the higher sophistication levels, to a Cohen’s *d* of .06 at the highest sophistication level.

**Fig 4:**
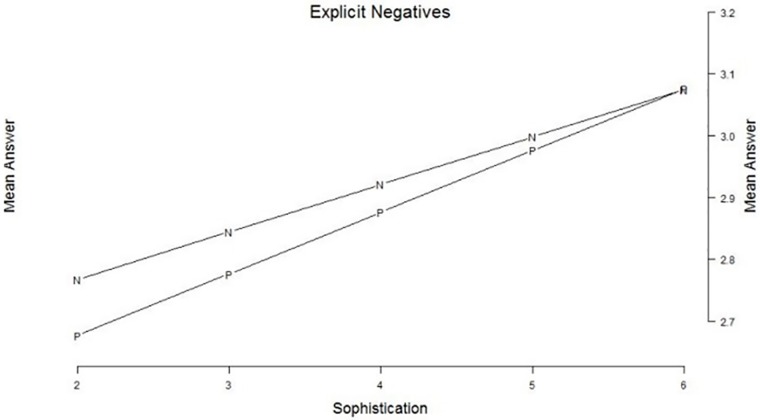
Political sophistication as a moderator of the explicit negatives. *Legend*: The answers are expressed on a 5-points scale ranging from a negative (1) evaluation of the attitude object to a positive evaluation (5). Sophistication is an additive scale combining education and political interest, expressed on a 5 points scale from low sophistication (2) to high sophistication (6).

For the set of 10 questions with explicit negations, we also find a significant interaction between political sophistication and question wording (*z* = 4.6, *p* < .001). Again, the direction of this interaction effect is similar to expectations from the literature: the higher the political sophistication, the smaller the wording effect of explicit negations (see Fig 4). Looking at the effect sizes in relation to the question variance, the wording effect is only really existant for the lowest sophistication group (Cohen’s *d* being .2 for sophistication level 2).

As a robustness check, we re-ran the above analyses also for the two different constituent variables of political sophistication, i.e. political interest and education. We find similar patterns for each variable separately, but these patterns only reach significance if education is used as a separate moderator.

## Discussion

In a field experiment during the Dutch municipal elections in the city of Utrecht in 2014, we investigated the effect of statement polarity on the answers obtained. Results show that the mean answers to questions with implicit negatives are affected by the question wording in the direction predicted based on previous research ([13], [14], [15], [16], [17], [18], [19]; Hypothesis 1a): overall, implicit negative questions lead to (slightly) more positive attitude reports compared to their positive counterparts. This overall pattern does not necessarily predict that each and every negative question will always lead to more disagreeing answers compared to its positive counterpart, as variation between questions is quite large.

This study is the first to show that political attitude questions with explicit negations lead to an overall wording effect in a similar direction to the implicit negatives: the answers to explicit negative questions suggest more positive evaluations of the attitude object than the answers to their positive counterparts, again with quite some variation in the direction of the wording effect between questions.

Our research shows that the question polarity effects are related to political sophistication, as put forward in Hypothesis 2: the higher the level of political sophistication, the smaller the effect of question wording on the answers. This is the first study to use sophistication as an operationalization of attitude strength in this type of political survey context, and results are in line with predictions from the literature in social psychology (e.g., [28]) as well as in survey methodology (e.g., [29]). This is the case for the set of implicit negative questions as well as for the set of questions containing explicit negations.

All in all, we find consistent effects confirming negativity bias: implicit as well as explicit negatives lead to an overall tendency to disagree, hence suggesting more positive evaluations of the attitude object in the question compared to when the positive equivalent question is asked. For implicit negatives, we find similar wording effects in this VAA context compared to findings reported on the forbid/allow asymmetry in attitude survey questions, this time for a wider range of implicit negative verbs (e.g., ‘*abolish’* vs. ‘*maintain’*, ‘*continue’* vs. ‘*stop’*). For explicit negations, this is the first study to show a general effect for this type of wording variation for two different types of explicit negations (‘*not’* and ‘*none’*) in political attitude questions. Furthermore, we find these effects are moderated by political sophistication in way that was predicted by the literature. However, the variation between questions in the size and directions of the effects is large; and the interaction effect with political sophistication shows that the overall wording effect is mainly caused by VAA users with a lower political sophistication. The implications for theory and practice are addressed below.

### Negativity

This is the first study to show an overall effect of negativity that is generalizable across sets of questions and across two types of negative word pairs in VAAs. Negativity bias ([24], [25]) describes that positive and negative contrasts do not necessarily carry the same weight: positives are used more often and reflect more moderate meanings compared to their negative counterparts. It may be that negative attitude statements are felt to be too strong to endorse (compared to answering disagreeing to their positive counterparts). It should be noted however that, similar to forbid/allow research reported in a survey context ([13], [20]), we do not find consistent wording effects for each and every question. Hence, there seems to be a complex interaction between the negativity of the evaluative term, and the context such as the issue in the question.

Interestingly, this variation might be explained from negativity bias as well, as negativity has a wider scope than just the words in the question. In a question ‘*polluting cars should be banned*’, the evaluative term ‘*banned*’ was expected to be too extreme to endorse, whereas it would be more easy to answer ‘*no*’ to the opposite positive question with the word ‘*allow*’. But if we zoom out to the content of the question, the attitude object ‘*polluting cars*’ is something that is generally evaluated negatively, and could hence give rise to an extreme response anyway. So, if linguistic negativity (‘*banned*’) is combined in one sentence with an attitude object that is conceptually negative, this may lead to subdued polarity effects. To complicate things further, some issues might instantly evoke positive evaluations with left wing voters, and negative evaluations with right wing voters (cf. [44]), which would imply we could expect an interaction between existing attitudinal negativity (or positivity) activated by concepts in the question (because of the user’s political background) and the polarity of the question. Future research into valence framing effects should look for a way to distinguish between (the effects of) linguistic, conceptual and attitudinal negativity.

### Motivated users?

Not only do we find quite some variation in the wording effects, we also observe effects in our VAA study that are somewhat smaller than the ones usually reported in the survey literature. These smaller effect sizes might be partly attributed to the five-points scales used in this VAA. Previous research in a survey context established smaller forbid/allow asymmetries when more fine-grained answering scales were offered compared to yes/no-scales ([13], [21]).

The smaller effects might also be attributed to the VAA context, as VAA users may generally be more motivated and probably show higher levels of political interest compared to respondents in a general political survey. A VAA differs from a regular opinion survey in that users visit the VAA spontaneously; they are motivated to fill it out and to think about their opinions more thoroughly than when filling out an attitude survey (e.g., [45]). This stronger motivation to think may have decreased shallow processing or satisficing behaviour, and may therefore have diminished the size of the question wording effects. To test this explanation further, it would be interesting to conduct a study in which the same positive and negative questions are compared in a different usage context.

Political sophistication is related to motivation. In our study, variation in users’ level of political sophistication was systematically related to the size or occurrence of wording effects. The higher the political sophistication, the smaller the overall wording effect—and the group of VAA users with the highest levels of political sophistication were not susceptible to the effects of question wording at all. This is an interesting result, in line with predictions about the role of attitude strength in survey methodology, as well as with more general theories on information processing and (political) opinion formation.

### Some methodological considerations

Voting Advice Applications are not only widely used tools in modern democracies, but they are also a very interesting context with high external validity to study the effect of wording variation. A strong advantage of doing this type of research in the context of VAAs rather than in surveys, is that the context of VAAs automatically implies many checks on the validity of the question wording variation, as not only the researcher, but also the VAA builder(s) and the politicians have to agree that a certain wording variation is relevant and valid in the political context of these specific elections. Furthermore, a large percentage of the electorate will use the VAA because they want to obtain a voting advice, causing these users to be motivated to think about the questions and use the instrument seriously. Our study showed that subtle question wording variation still affects the answers—even in this usage context of likely deep processing.

While we conducted our experiment in the context of local elections in the city of Utrecht, the process of VAA construction here was comparable to the construction of VAAs in other municipalities or during different local elections in the Netherlands. In addition, as the demographic characteristics of our VAA users match the typology of VAA users generally established in VAA research (i.e., [40]), our results can be extrapolated to the general population of VAA users too. And finally, because VAA users were randomly assigned to conditions the current study also has a high internal validity.

### Implications

The increasing popularity of VAAs in many democracies stresses the relevance of research into the possible impact of seemingly small design choices in these ‘political decision aids’. The effects of question wording on VAA answers corroborate the idea that VAAs not only inform of the debate during elections, but also contribute to constructing the content of the discussion [3]. The effect of implicit and explicit negative question wordings on the answers to VAA questions suggests that this wording variation may also affect the voting advice based on these answers, and might thus affect the users’ perception of their position in the political landscape, as well as affect their opinion formation towards the issues in the VAA. The current research suggests this holds most specifically for the users with lower levels of political sophistication.

Future research might want to address the extent to which question wording variation in VAAs not only affects the answers, but also influences the resulting voting advice. A difficulty in modelling this, however, is that it would be unethical in a field study to construe a VAA version with negative (or positive) questions only.

VAAs aim to reach all voters, and also reach out to the less sophisticated voters, as they have most to gain in terms of political knowledge and political efficacy by using a Voting Advice Application. This research shows that VAA builders should be careful in wording the questions in their Voting Advice Applications. Which question wording they should prefer, cannot be advised on conclusively based on this research, however. A next useful step could be to investigate how the party positions for each VAA statement, as established by professional coders working for a VAA builder, are affected by the positive or negative wordings of the questions. If party positions are affected differently by variation in the question wording than VAA users’ answers, the validity of the voting advice is threatened more strongly compared to a situation in which party positions and VAA users’ answers shift in similar ways.

All in all, the present study shows convincingly how the choice of wording in an online mass medium such as a VAA contributes to public opinion in election time. It is crucial to deepen our understanding of public opinion formation through more research on the way subtle design characteristics of VAAs affect the answers citizens give.

## Appendix 1: Model Specifications

In Eq 1, the model used for analyzing effect of question polarity on the mean substantive answer for the set of implicit linguistic contrasts is formalized. In this model, *Y*_*(jk)*_ indicates the answer of individual *j* (*j* = 1, 2…31,112) to question *k* (*k* = 1, 2,…6), which is the dependent variable we aim to predict. In the model, a constant is used to estimate the mean answer to positive questions (CONS β_1_), which may vary between persons (*u*_*j*0_), questions (*v*_0*k*_), and the interaction between the two (*e*_*(jk)*_). Next the model adds a fixed parameter to estimate how the mean answer to negative questions deviates from the mean for positive questions Deviation_Neg (β_2_). The model assumes that the residuals are normally distributed, with an expected value of zero, and a variance of respectively S^2^_*e*1(*jk*)_, S^2^_*u*1*j*0,_ S^2^_*v*0*k*_.

$$Y_{\left(jk\right)}=CONS_{\left(jk\right)}\left(\beta_{1}\right)+D\_Neg_{\left(jk\right)}\left(\beta_{2}\right)+\left[e_{1\left(jk\right)}+\;u_{1j0}+v_{0k}\right]$$ (1)

The second model adds the main effect of political sophistication as well as the interaction between question polarity and political sophistication. This is shown in Eq 2.

$$\begin{array}{l} Y_{\left(jk\right)}=CONS_{\left(jk\right)}\left(\beta_{1}\right)+D\_Neg_{\left(jk\right)}\left(\beta_{2}\right)+\;Political\_sophistication_{\left(j0\right)}\left(\beta_{3}\right)\\ +D\_Neg\times Political\_sophistication{\;}_{\left(j0\right)}\beta_{4}+\left[e_{1\left(jk\right)}+u_{1j0}+v_{0k}\right] \end{array}$$ (2)

## Supporting Information

S1 Appendix Manipulated positive and negative statements and their rough English translation. (PDF)
