# Supplementary material for: Positive vs. Negative: The Impact of Question Polarity in Voting Advice Applications
Source: PLoS One. 2016 Oct 10;11(10):e0164184. doi: 10.1371/journal.pone.0164184 (PMC5056712; doi:10.1371/journal.pone.0164184)
Supplement: S1 Appendix — (PDF) [file pone.0164184.s001.pdf]

SI Appendix.

Manipulated positive and negative statements and their rough English translation.

Statement order was kept constant across versions. Between brackets, right after the Dutch question wording, the versions are indicated in which this wording was included. Following to each question number, the code IM or EX indicates whether the question belongs to the group of Implicit Negatives or Explicit Negatives.

No.	Positive wording	Negative wording	English literal translation of the positive wording	English literal translation of the negative wording
1 EX	Er moet een verbreding komen van de A27 bij Amelisweerd (A + C + D)	Er moet geen verbreding komen van de A27 bij Amelisweerd (B + E)	The A27 highway should be broadened at Amelisweerd	The A27 highway should not be broadened at Amelisweerd
2 EX	De parkeertarieven in Utrecht mogen worden verhoogd (A + B + E)	De parkeertarieven in Utrecht mogen niet worden verhoogd (C + D)	Parking charges should be raised in the city of Utrecht	Parking charges should not be raised in the city of Utrecht
3 IM	De meest	De meest	The cars	The cars

	vervuilende auto's (ouder dan Diesel Euro 3 en Benzine Euro 0) moeten in de binnenstad worden toegelaten (D + E)	vervuilende auto's (ouder dan Diesel Euro 3 en Benzine Euro 0) moeten uit de binnenstad worden geweerd (A + B + C)	polluting most (older than EURO 3 for diesel and EURO 0 for gasoline) should be allowed in the city center	polluting most (older than EURO 3 for diesel and EURO 0 for gasoline) should be banned from the city center
4 IM	Het budget om de leefbaarheid in wijken te verbeteren (het leefbaarheidsbudget) moet worden gehandhaafd (B + C)	Het budget om de leefbaarheid in wijken te verbeteren (het leefbaarheidsbudget) moet worden afgeschaft (A + D + E)	The budget for improving neighbourhood liveability (the liveability budget) should be maintained	The budget for improving neighbourhood liveability (the liveability budget) should be abolished
5 EX	In achterstandswijken mogen sociale huurwoningen worden gesloopt (A + B + E)	In achterstandswijken mogen geen sociale huurwoningen worden gesloopt (C + D)	In disadvantaged neighborhoods, the municipality may tear down	In disadvantaged neighborhoods, the municipality may not tear down social

			social housing	housing
6 EX	Op welzijnswerk mag worden bezuinigd (A + C + D)	Op welzijnswerk mag niet worden bezuinigd (B + E)	The municipality can cut down on social work	The municipality cannot cut down on social work
7 EX	Er mogen woningen worden gebouwd in de polder Rijnenburg (A + B + E)	Er mogen geen woningen worden gebouwd in de polder Rijnenburg (C + D)	The municipality can build houses in the Rijnenburg polder	The municipality can build no houses in the Rijnenburg polder
8 EX	De gemeente moet geld investeren in een fietsbrug tussen Oog in Al en Leidsche Rijn over het Amsterdam-Rijnkanaal (A + D + E)	De gemeente moet geen geld investeren in een fietsbrug tussen Oog in Al en Leidsche Rijn over het Amsterdam-Rijnkanaal (B + C)	The municipality should invest money to build a bicycle bridge over the Amsterdam-Rijn canal between Oog	The municipality should not invest money to build a bicycle bridge over the Amsterdam-Rijn canal between Oog in Al and Leidsche Rijn

			in Al and Leidsche Rijn	
9 EX	De gemeente moet extra geld investeren om taalachterstanden bij kinderen tegen te gaan (A + C+ D)	De gemeente moet geen extra geld investeren om taalachterstanden bij kinderen tegen te gaan (B + E)	The municipality should invest extra money to fight children's language deficiencies	The municipality should not invest extra money to fight children's language deficiencies
10 EX	Op kunst en cultuur mag worden bezuinigd (A + B + C)	Op kunst en cultuur mag niet worden bezuinigd (D + E)	The municipality may cut down on art and culture	The municipality may not cut down on art and culture
11 EX	Voor milieumaatregelen mag een belastingverhoging plaatsvinden (A + B + E)	Voor milieumaatregelen mag geen belastingverhoging plaatsvinden (C +D)	Taxes may be increased for measures for the environment	Taxes may not be increased for measures for the environment
12 IM	De gemeente moet bedrijven zelf laten bepalen of ze	De gemeente mag bedrijven dwingen tot het nemen van	The municipality should let	The municipality may force businesses to

	energiebesparende maatregelen nemen (B+C)	energiebesparende maatregelen (A + D+ E)	businesses decide for themselves whether they take energy-saving measures	take energy-saving measures
13 EX	Op industrieterrein Lage Weide mag een windmolenpark komen (A + C + D)	Op industrieterrein Lage Weide mag geen windmolenpark komen (B + E)	A wind park can be built at the Lage Weide industrial zone	No wind park can be built at the Lage Weide industrial zone
14 IM	De verplichte vergunning voor het verbouwen van een eigen huis moet worden gehandhaafd (C + E)	De verplichte vergunning voor het verbouwen van een eigen huis moet worden afgeschaft (A + B + D)	The requirement for a building permit of one's own house should remain to exist	The requirement for a building permit of one's own house should be abolished
15 IM	Circussen met dieren moeten in Utrecht toegelaten blijven (C + D)	Circussen met dieren moeten in Utrecht verboden worden (A + B + E)	Circuses with animals should remain allowed in Utrecht	Circuses with animals should be forbidden in Utrecht

16 IM	De ontwikkeling van een gemeentelijke wietkwekerij moet worden doorgezet (C + E)	De ontwikkeling van een gemeentelijke wietkwekerij moet worden gestopt (A + B + D)	The development of municipal marihuana nursery should be continued	The development of municipal marihuana nursery should be stopped

Note. The experiment also included questions on demographic variables as well as filler VAA statements. The full experimental materials are available on demand. The experiment (in Dutch) can be accessed via https://utrecht2014.kieskompas.nl/
